# Studying the Biological Activity of Cerium Dioxide Nanoparticles Using Bacterial Biosensors

**DOI:** 10.3390/ijms27073179

**Published:** 2026-03-31

**Authors:** Ekaterina V. Silina, Evgeniya V. Prazdnova, Sergey A. Emelyantsev, Ludmila E. Khmelevtsova, Varvara N. Statsenko, Natalia E. Manturova, Kseniia A. Palkina, Ilia V. Yampolsky, Victor A. Stupin

**Affiliations:** 1 Institute of Biodesign and AI in medicine, I.M. Sechenov First Moscow State Medical University (Sechenov University), 119991 Moscow, Russia; 2 Academy of Biology and Medicine named after D.I. Ivanovskiy, Southern Federal University, 344090 Rostov-on-Don, Russia; emelyancev@sfedu.ru (S.A.E.); lehmelevcova@sfedu.ru (L.E.K.); varvarastac@mail.ru (V.N.S.); 3 Department of Surgery, Pirogov Russian National Research Medical University (RNRMU), 117997 Moscow, Russia; manturovanatali@yandex.ru (N.E.M.); stvictor@bk.ru (V.A.S.); 4 Shemyakin-Ovchinnikov Institute of Bioorganic Chemistry, Russian Academy of Sciences, 117997 Moscow, Russia; palkina_1993@mail.ru (K.A.P.); ivyamp@gmail.com (I.V.Y.)

**Keywords:** biosensors, nanoparticles, cerium, biotesting, toxicity, nanotoxicology, antioxidants, molecular microbiology, biosensing techniques, *Escherichia coli*

## Abstract

Cerium oxide nanoparticles (CeO_2_NPs) possess unique physicochemical properties that make them promising compounds for medical and industrial applications. However, variations in synthesis methods, particle size, and surface characteristics may influence their potential toxicity. This study provides a comparative analysis of CeO_2_NPs synthesized via three methods (citric, dextran, and uncoated modifications) to evaluate their toxicity, antioxidant mechanisms, and genoprotective potential using a panel of *Escherichia coli*-based lux-biosensors. Our data indicate that all of the tested CeO_2_NPs exhibit high biocompatibility with no significant toxicity or genotoxicity at physiological concentrations (10^−4^–10^−2^ M). The citrate-modified nanoparticles demonstrated pronounced catalase-mimetic activity, acting as the most effective scavengers against hydrogen peroxide. Conversely, the dextran-modified nanoparticles exhibited the highest antimutagenic potential, reducing dioxidine-induced DNA damage by over 56%. Thus, beyond establishing biocompatibility, this study highlights the potential of using specific CeO_2_NP modifications for targeted therapy depending on the oxidative pathway involved. This suggests their potential for application as antioxidant and antimutagenic agents in both human and veterinary medicine.

## 1. Introduction

Nanomaterials represent a rapidly developing and promising field of research, as they possess unique physicochemical properties and have a wide range of applications. In recent years, research on cerium oxide nanoparticles (CeO_2_NPs) has shown promising results in biomedical applications due to their antioxidant and anticancer properties [1,2]. The high surface area-to-volume ratio of nanocerium is critical in the formation of oxygen defects (called reactive sites) in its structure, which can be used as free radical scavenging sites [3]. Due to their large number of reactive sites, CeO_2_NPs exhibit long-lasting antioxidant activity, which distinguishes them from classical antioxidants [4]. Most antioxidants become depleted in the process of reactive oxygen species (ROS) scavenging, but cerium oxide can cycle between the Ce^3+^ and Ce^4+^ oxidation states. CeO(III) is rapidly oxidized to CeO(IV) by ROS, and thus CeO(III) successfully scavenges ROS. CeO(IV) is slowly reduced again by ROS, reverting to CeO(III) and leaving oxygen vacancies in the CeO crystal lattice, which becomes even more reactive toward ROS. Thus, the cyclic switching of CeO between the 3+ and 4+ states can continuously exhaust ROS [5,6,7,8], so CeO_2_NPs hold promise for the development of drugs for the treatment of a wide range of diseases associated with oxidative stress, which is of great importance for several somatic diseases [9,10,11]. Moreover, these effects were repeated when using several forms containing nanocerium. For example, polyethylene glycol (PEG)-based nanocerium has been shown to improve and accelerate wound healing in rat studies [12]. Dextran-coated nanoceria, citrate-coated nanoceria, and uncoated nanoceria, synthesized from cerium(III) nitrate aqueous solution at a nanoparticle concentration of 10^−3^ M and a size of up to 10 nm, also showed wound healing properties, stimulating the proliferation and metabolism of human cell cultures involved in wound repair (mesenchymal stem cells, keratinocytes, and fibroblasts) up to 2.5-fold [13,14,15,16,17,18]. In a systematic review, Xue et al. [19] summarized information on various forms of CeO_2_ nanoparticles that have been used in wound healing materials over the past decade. In addition, nanocerium also shows promise in the treatment of somatic diseases not associated with common etiopathogenetic roots [17,18,19,20,21,22,23,24,25].

At the same time, there are concerns that the presence of nanoceria in the human body, either through their use as a drug or because of industrial pollution, could have negative effects. Data on possible negative effects were collected through years of studying CeO_2_ nanoparticles with various coatings, sizes, concentrations, and other physical factors [26,27,28,29]. Depending on their size, aggregation, and synthesis method, nanoparticles can exhibit both antioxidant and prooxidant properties [4,25,28]. Given their ability to penetrate cell membranes and their potential impact on nuclear DNA, one could suggest that these particles may cause mutagenic effects [30,31,32,33]. Thus, it is crucial to continue studying the potential toxicity of CeO_2_ nanoparticles and the mechanisms underlying it to avoid adverse effects from CeO_2_ use.

For a long time, toxicity and oxidative stress assessment was conducted using several well-known methods: (1) Chemical or physical methods—these evaluate only indirect chemical reactions that are loosely related to the complex life of a living organism; (2) Cell culture methods—these evaluate the responses of specific types of differentiated or undifferentiated progenitor cells, which can vary [26]; and (3) Laboratory animal studies—these increase the cost and cumbersome nature of the study and raise ethical concerns [34].

Therefore, increased research is being conducted on relatively simple and easy-to-maintain living organisms. Most often, toxicity to living organisms is studied using model objects such as the crustacean *Artemia salina* [34,35], the nematode *Caenorhabditis elegans* [36,37], the aquatic midge *Chironomus riparius* [37], the microalgae *Pseudokirchneriella subcapitata* [38], and the earthworm *Eisenia fetida* [39].

Biotesting using bacterial biosensors may be a good alternative to the use of animals [40]. Bacteria adapt quickly to their environment, allowing them to detect and respond to environmental changes such as fluctuations in chemical concentrations and temperature [41]. This makes bacteria some of the most suitable biological reporters that convert corresponding chemical signals into a readable signal. Bacterial biosensing is typically two-pronged: biological sensing elements (microbial cells) that can selectively identify the target chemical and produce signals related to its concentration, and physical devices that convert and detect optical, electrochemical, and thermodynamic signals [42]. The advantages of using bacterial biosensors include low cost, speed of analysis, sensitivity, and the ability to detect not only the presence or absence of toxicity, but also to suggest the mechanism by which it occurs (damage to DNA, proteins, membranes, oxidative stress, etc.) [43]. Previously, we successfully and repeatedly used bacterial lux-biosensors based on *E. coli* to study the positive and negative biological properties of both newly synthesized substances and natural compounds including nanoparticles [40,44,45]. The biosensor strains used in these studies harbored bacterial luciferase genes under the control of bacterial promoters specifically induced by various chemicals or cellular stress.

The aim of this study was to investigate the biological effects of cerium oxide nanoparticles (CeO_2_NPs), synthesized by three different methods (C-1—citric nanocerium; C-2—dextran nanocerium; and C-3—uncoated nanocerium), using bioassay methods and a set of bacterial lux-biosensors to determine their overall toxicity, pro-/antioxidant status, and pro-/antimutagenic properties in bacteria.

This study used three types of CeO_2_NPs obtained with different methods: 1—precipitation from aqueous solutions of cerium(III) nitrate hexahydrate and citric acid in a 1:1 ratio, with the size of the synthesized nanoparticles being mainly 3–4 nm [14,15,17]; 2—using the same cerium nitrate and dextran formulation at a 1:2 initial ratio (Ce2D, with a particle size of mainly 1–3 nm) [16,17]; and 3—the thermal decomposition of cerium carbonate obtained by precipitation from nitrate aqueous solution (the best fraction of the CeO_2_-II sample was used, which had a particle size of up to 10 nm (mainly 6–9 nm)) [13]. The choice of these types and sizes of CeO_2_NPs and the concentrations studied were based not only on previously obtained positive results, but also on the results of a systematic review proving that CeO_2_NPs with sizes up to 10 nm at doses ≤ 5 mg/kg show no signs of toxicity [46].

## 2. Results

### 2.1. Cerium Oxide Nanoparticle Characteristics

**Figure 1:**
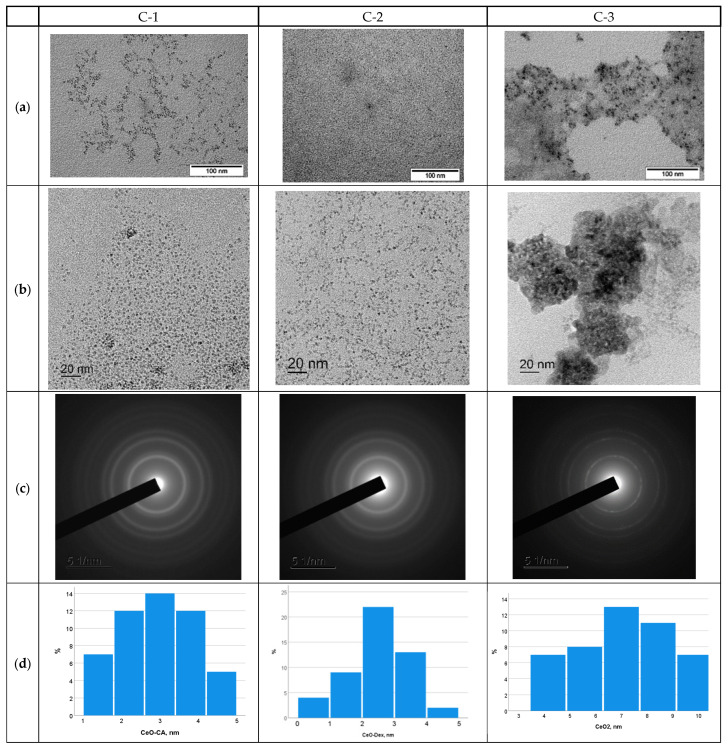
TEM images of CeO_2_ C-1 (citric nanocerium), C-2 (dextran nanocerium), and C-3 (uncoated nanocerium). (**a**) Overview image; (**b**) enlarged image (ruler size 20 nm); (**c**) electronogram; (**d**) particle size distribution. On TEM, the organic coat is not visible, only the core of the nanoparticles.

Figure 1 shows the transmission electron microscopy (TEM) micrographs and electronograms of three variants of CeO_2_ nanoparticles.

The overview and magnified microimages of TEM samples C-1 and C-2 show that the nanocerium particles stabilized by citrate and dextran were smaller than 5 nm. Sample C-3 (nanocerium without a stabilizing coating) had a high tendency to agglomerate, which highlights the advisability of using nanoparticle stabilizing excipients. The particles that made up the agglomerate were finely dispersed and crystalline. Chaotically oriented nanocrystallites of 6–10 nm size were visible in the agglomerate, and individual non-polymerized finely dispersed nanoparticles <5 nm were also present. The location of the diffraction maxima on the annular electrogram of all three samples (Figure 1c) corresponded to the phase CeO_2_. The blurriness of the rings in the electron diffraction patterns also indicates the finely dispersed structure of the nanoceria samples. As can be seen from the nanoparticle distribution data (Figure 1d), the average crystallite size was 2.9 ± 0.9 nm (mostly 2–4 nm) for sample C-1, 2.4 ± 0.8 nm for C-2 (mostly 2–4 nm), and 7.0 ± 2.1 nm for C-3 (mostly 6–9 nm).

**Figure 2:**
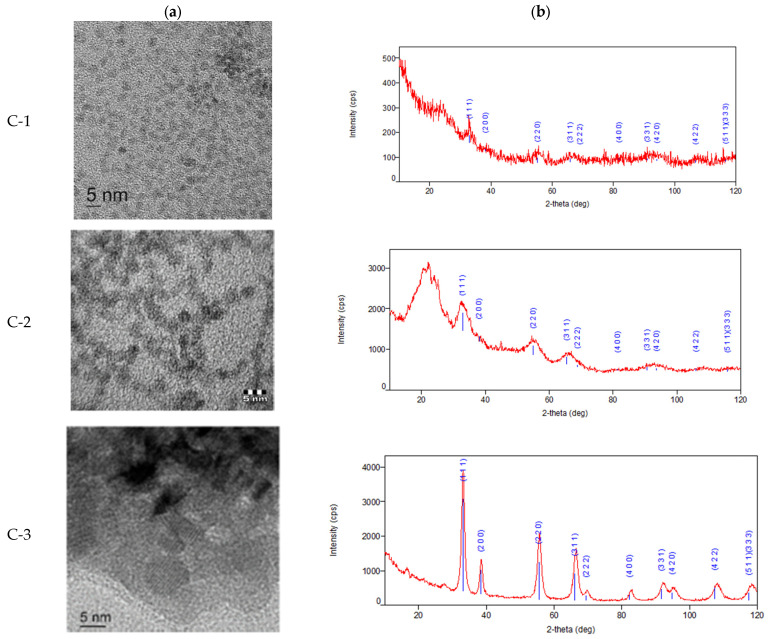
Characterization of nanoparticles after synthesis: C-1 (citric nanocerium), C-2 (dextran nanocerium), and C-3 (uncoated nanocerium). (**a**) TEM images of CeO_2_NPs at maximum magnification (ruler size 5 nm); (**b**) X-ray diffraction. The data were partially published [13,14,15,16].

The resulting data obtained 12 months after the synthesis of nanoparticles were well-aligned with the previously obtained TEM and X-ray diffraction findings in our previous studies [13,14,15,16] (Figure 2). The results of the X-ray diffraction analysis (Figure 2b) indicate the presence of only one phase in the sample: cerium(IV) oxide with a face-centered cubic lattice (space group Fm-3m). The size of the coherent scattering region was on average 2.7 (0.4) nm for C-1, 2.4 (0.5) nm for C-2, and 9.1 (3) nm for C-3.

**Figure 3:**
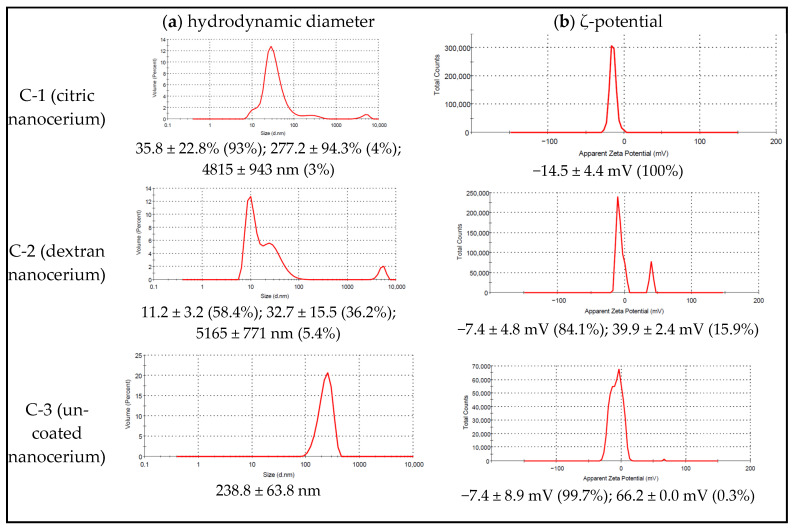
Dynamic light scattering data for cerium dioxide samples C-1, C-2, and C-3, examined 12 months after synthesis: the particle sizes with hydrodynamic diameter (**a**) and ζ-potential (**b**).

The results of dynamic light scattering are presented in Figure 3.

In sample C-1, individual particles were found to be 35 nm (10–60 nm) in size (93%) and to form aggregates ranging from 100 nm to 6000 nm in diameter at a low ζ-potential (|15| mV). 

In sample C-2 (dextran nanocerium), individual particles of size 8–15 nm (58% of NPs) and aggregates of 20–50 nm diameter (36%) were detected. Large agglomerates with an average size of 5165 nm at 5% were also identified. The native sample had a ζ-potential of −7.4 mV (84%) and 40 mV (16%). 

According to the results of dynamic light scattering, the colloidal solution C-3 contained agglomerates of 100–300 nm at a low ζ-potential (7.4 ± 8.9 mV), indicating instabilities in nanoparticles.

### 2.2. CeO_2_NP Toxicity Assessment Using Biosensor Test

The abbreviated names of the cerium nanoparticle samples and their concentrations are presented in Table 1.

#### 2.2.1. Fluorescent Biosensors

**Figure 4:**
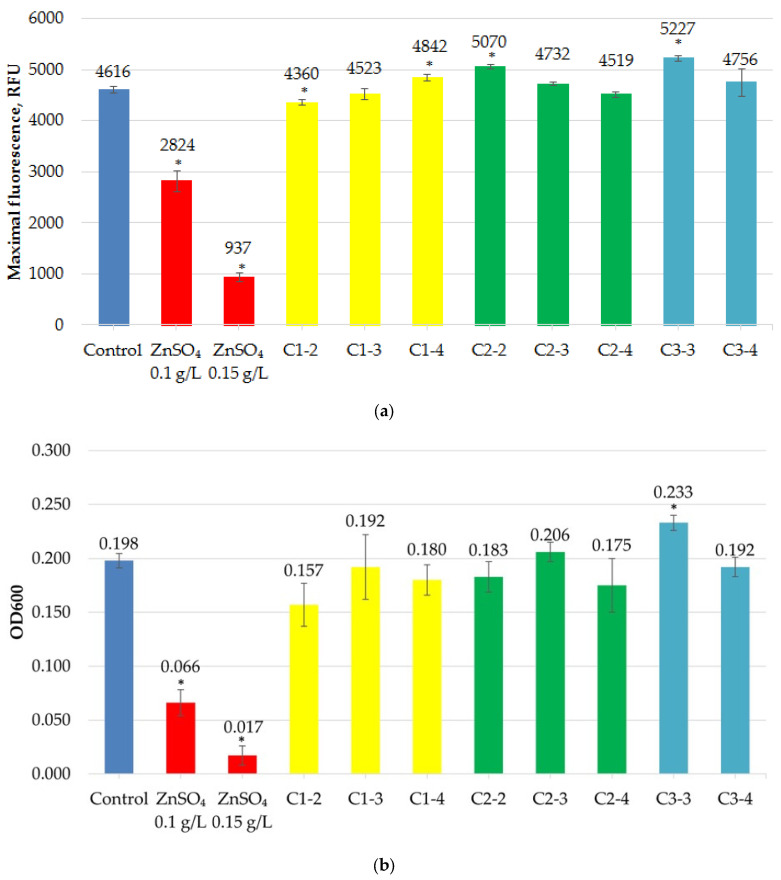
Maximum fluorescence (**a**) and optical density at 600 nm (OD600) (**b**) of the *E. coli* XL1-Blue eGFP strain in the presence of CeO_2_NPs. “*” indicates statistically significant differences from the control (two-tailed Student’s *t*-test, Bonferroni-corrected *p* < 0.05).

Incubation of the fluorescent *E. coli* strain XL1-Blue eGFP with cerium nanoparticles demonstrated no significant negative effect on fluorescence (Figure 4a) or bacterial culture growth (Figure 4b). Maximum fluorescence and OD600 values were selected over 120 min of incubation. The degrees of fluorescence suppression and bacterial culture growth in the presence of nanoparticles were assessed after 120 min in the experiment, as this is when the culture density typically peaks.

Some samples induced fluorescence insignificantly, but a statistically significant suppression of fluorescence was observed only in the C1-2 sample. A significant, albeit slight, decrease in culture density was also observed only in the C1-2 sample.

The percentage of fluorescence inhibition was 82.4 ± 1.8% for 0.15 g/L zinc sulfate and 43.4 ± 5.3% for 0.1 g/L zinc sulfate (positive controls). For the test substances, it ranged from 0 to 12.6%, with a maximum value of 12.6 ± 0.9% observed for sample C1-2.

The percentage of growth inhibition (turbidity) was 95.5 ± 7.2% for 0.15 g/L zinc sulfate and 70.4 ± 6.3% for 0.1 g/L zinc sulfate (positive controls). For the test substances, it ranged from 0 to 20.7%, with a maximum value of 20.7 ± 3.4% observed for sample C1-2.

It can be concluded that none of the studied samples significantly inhibited fluorescence, and only one inhibited the growth of the *E. coli* XL1-Blue eGFP strain.

**Figure 5:**
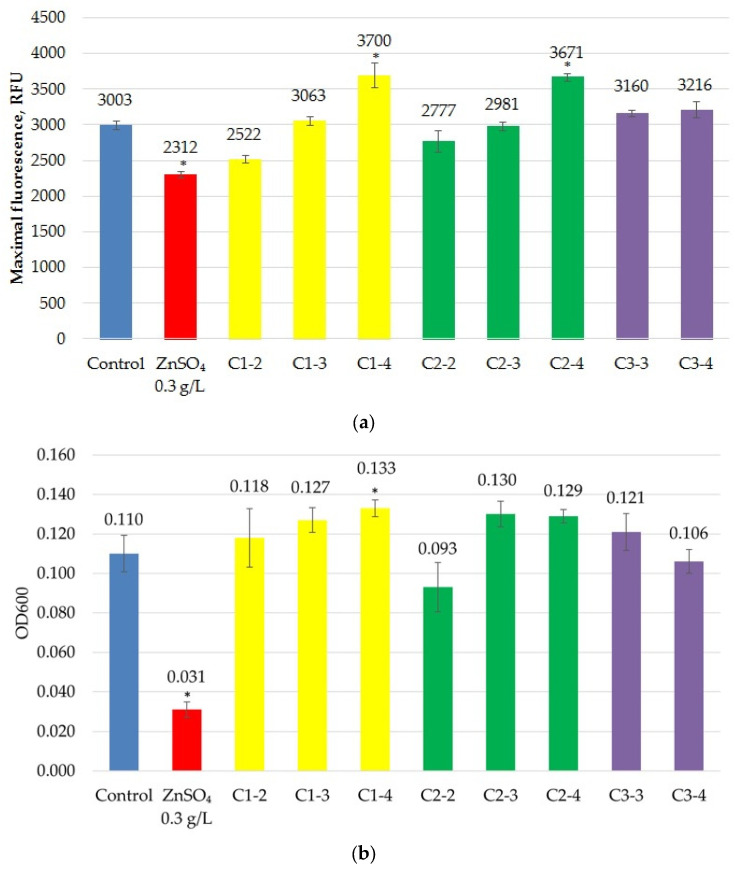
Maximum fluorescence (**a**) and optical density at 600 nm (OD600) (**b**) of *E. coli* XL1-Blue FusionRed in the presence of CeO_2_NPs. “*” indicates statistically significant differences from the control (two-tailed Student’s *t*-test, Bonferroni-corrected *p* < 0.05).

When testing cerium oxide nanoparticles with another fluorescent strain, *E. coli* XL1-Blue FusionRed, a slight stimulating effect on bacterial culture fluorescence (Figure 5a) and growth (Figure 5b) was observed for most of the samples.

However, sample C1-2 (citrate cerium nanoparticles at the maximum studied concentration) demonstrated the ability to suppress fluorescence (by 16 ± 1.5%), and sample C2-2 also inhibited culture growth by 19 ± 2.7%, although this effect was not statistically significant (*p* > 0.05).

Moreover, the fluorescence suppression rate was 23 ± 3.2%, and the growth suppression rate (turbidity) was 87.8 ± 7.1% for 0.3 g/L zinc sulfate (positive control).

Thus, testing with fluorescent biosensors showed that only cerium citrate at a concentration of 10^−2^ M exhibited mild toxicity.

#### 2.2.2. Luminescent Biosensors

**Figure 6:**
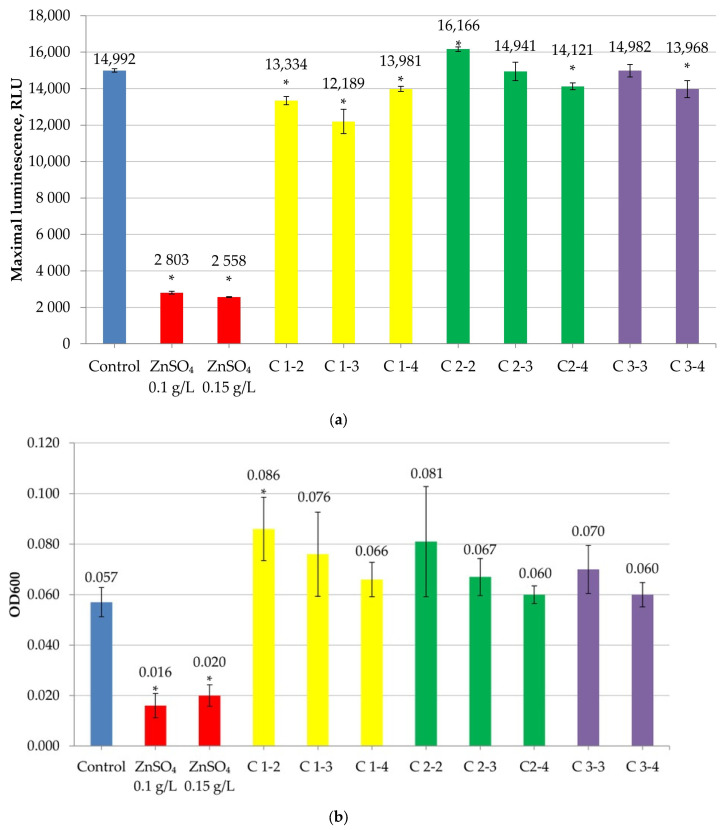
Maximum luminescence (**a**) and density (**b**) of *E. coli* XL1-Blue pNK3751 in the presence of CeO_2_NPs. “*” indicates statistically significant differences from the control (two-tailed Student’s *t*-test, Bonferroni-corrected *p* < 0.05).

The luminescence of *E. coli* XL1-Blue pNK3751 decreased slightly when incubated with cerium citrate nanoparticles at all concentrations studied (Figure 6a). Samples C1-2 suppressed fluorescence by 17.7 ± 2.6%, C1-3 by 24.8 ± 3.5%, and C1-4 by 13.8 ± 1.0% relative to the control. The “dextran” and “uncoated” cerium nanoparticle samples had no significant effect on the luminescence levels, with one exception: sample C2-2 slightly stimulated luminescence. None of the studied nanoparticles had an inhibitory effect on the growth of the *E. coli* XL1-Blue pNK3751 bacterial culture (Figure 6b).

A possible mechanism underlying the bioluminescence stimulation observed for the constitutive biosensor *E. coli* XL1-Blue pNK3751 in the dextran-coated sample C2-2 at the highest concentration (10^−2^ M) is that dextran, as a glucose polymer, serves as both a carbohydrate source for *E. coli* and as a substrate for luciferase oxidation.

In contrast, citric nanocerium slightly suppressed luminescence, which may result from a decrease in the medium’s pH caused by citrate, thereby reducing luciferase enzyme activity. Another potential inhibitory mechanism of citrate involves the chelation of metal ions in the bacterial cell wall, leading to the diminished uptake of essential nutrients from the medium [47].

**Figure 7:**
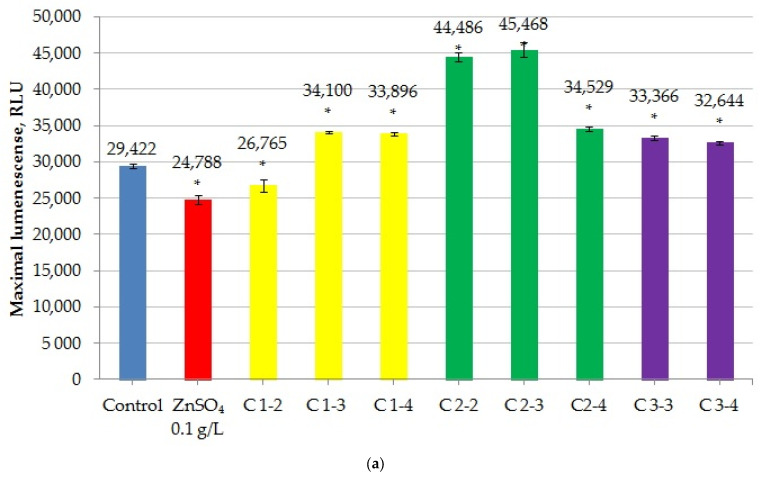

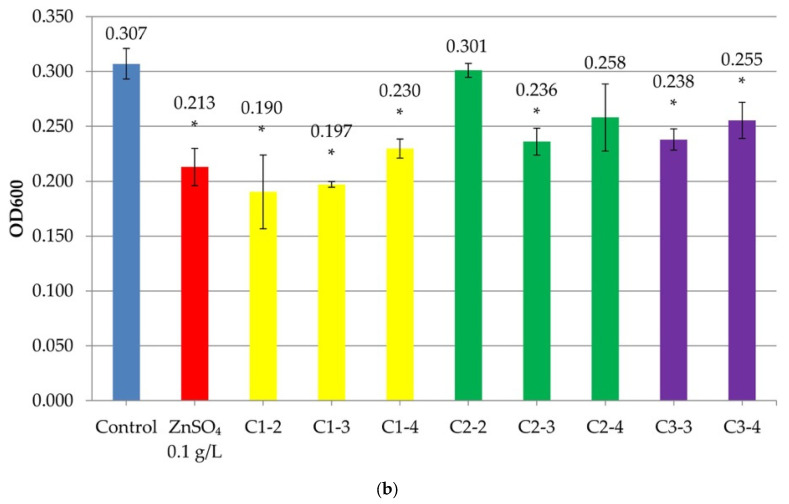
Maximum luminescence (**a**) and density (**b**) of the *E. coli* strain MG 1655 pXen7-lux in the presence of CeO_2_NPs. “*” indicates statistically significant differences from the control (two-tailed Student’s *t*-test, Bonferroni-corrected *p* < 0.05).

Similar results were obtained using another strain with constitutive luminescence: *E. coli* MG 1655 (pXen7-lux). All samples induced bacterial luminescence slightly except for high-concentration cerium citrate (C1-2, 10^−2^ M), which suppressed it (Figure 7a).

Almost all of the “citrate” cerium nanoparticles exhibited a slight bacteriostatic effect, reducing the optical density of the *E. coli* MG 1655 (pXen7-lux) bacterial culture compared to the control (Figure 7b). Sample C3-3 also demonstrated this effect.

The maximum bioluminescence inhibition of the *E. coli* strain MG 1655 (pXen7-lux) by CeO_2_NPs was 17.14 ± 2.25% (for sample C1-2), although the difference was not statistically significant. Meanwhile, inhibition by ZnSO_4_ was 15.75 ± 1.03% in the same test. However, significant growth inhibition with CeO_2_NPs was demonstrated in samples C1-3, C1-4, and C3-3 (34.63 ± 0.76%, 24.46 ± 2.78%, and 23.50 ± 3.13%, respectively), while ZnSO_4_ inhibited growth by 30 ± 8.5%.

The results demonstrate that 50% inhibition of culture growth, as measured by OD_600_ or complete luminescence inhibition, was not reached at any concentration of cerium oxide nanoparticles. Consequently, the minimum luminescence inhibition concentration (MLIC) was also not reached at any tested concentration.

Thus, testing the cerium nanoparticle samples using fluorescent and luminescent bacterial biosensor strains showed that, overall, most of the samples did not exhibit significant toxicity. The highest concentration of cerium citrate tested (10^−2^ M (C1-2)) demonstrated a suppressive effect on the emission of all biosensors (both fluorescence and luminescence). The biosensors also showed a slight bacteriostatic effect for some of the CeO_2_ nanoparticles tested.

### 2.3. Prooxidant Activity of CeO_2_NPs

Prooxidant activity was assessed based on the ability of the tested CeO_2_NP samples to induce or increase luminescence in strains with stress-inducible promoters. Antioxidant activity was assessed based on the ability of the samples to reduce the induction caused by the test inducer and was calculated as a percentage of induction suppression. The effects were tested using two models: hydrogen peroxide and methyl viologen, a superoxide anion radical generator.

**Figure 8:**
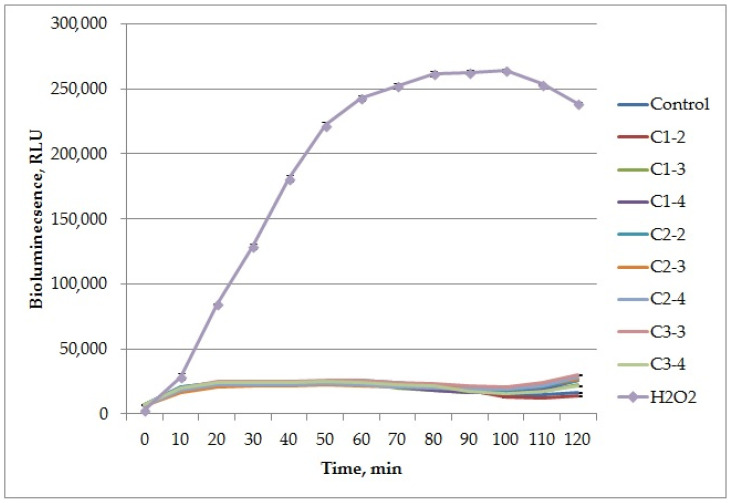
Effect of the studied CeO_2_NP samples on the bioluminescence of the *E. coli* biosensor strain MG1655 p(KatG-lux). H_2_O_2_ curve significantly differed from the control (two-tailed Student’s *t*-test, Bonferroni-corrected *p* < 0.05) in the timeframe of 10–120 min.

When testing cerium nanoparticles using the *E. coli* biosensor strain MG1655 (pKatG-lux), it was shown that none of the tested nanoparticle samples, at any concentration, exhibited a prooxidant effect arising from their ability to generate peroxides (Figure 8, Table 2).

Testing using the lux-biosensor *E. coli* strain MG1655 (pSoxS-lux) revealed the absence of prooxidant (superoxide anion generation) properties in the studied nanoparticles (Table 3).

In both tests, in no sample did the induction factor exceed 2. This value is considered the threshold for toxicity in biosensor tests on these strains, based on genotoxicological studies [48].

Thus, it can be concluded that the studied samples do not possess prooxidant activity, and that this activity is not affected by the presence of coatings, nanoparticle size, or concentration, within the limits of this study.

### 2.4. Antioxidant Activity of CeO_2_NPs

**Figure 9:**
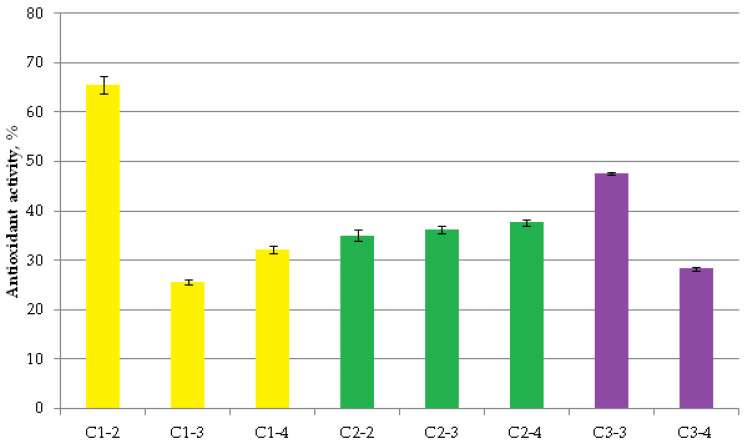
Antioxidant activity of CeO_2_NPs against hydrogen peroxide. All values significantly differed from zero, with zero defined as the absence of protective activity (*p* < 0.05, Student’s *t*-test).

When studying the ability of the synthesized cerium nanoparticles to protect cells from oxidative stress, it was found that the studied samples possessed statistically significant antioxidant activity against peroxide, although in most cases, this was to a lower level than that of model antioxidants. The model antioxidant in this test system is typically considered to be α-tocopherol, exhibiting approximately 70% activity, or ionol, with activity up to 93% [49]. Sample C1-2 was the most active, with an activity of 65.6% and the highest concentration of cerium citrate nanoparticles (Figure 9). The C3-3 sample was the second most potent antioxidant. The minimum protective activity (25.7%) against peroxide was exhibited by a sample of the same “citrate” cerium diluted by 10 times (Cl-3, 10^−3^ M).

**Figure 10:**
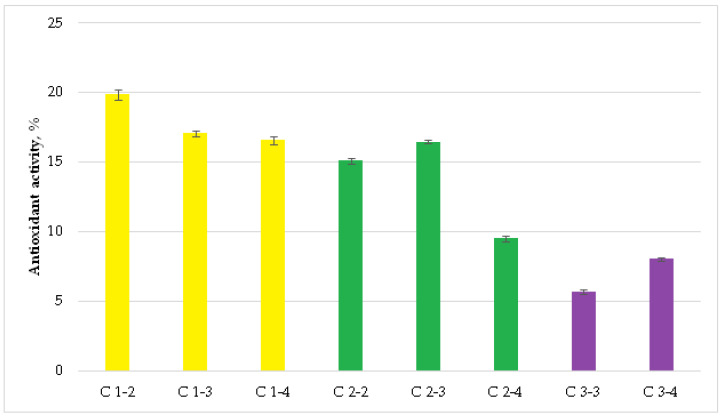
Antioxidant activity of CeO_2_NPs against methyl viologen (a superoxide anion radical generator). All values significantly differed from zero (indicating no protective activity) (two-tailed Student’s *t*-test, Bonferroni-corrected *p* < 0.05).

The antioxidant activity of CeO_2_NPs against the superoxide generator (methyl viologen) was low (Figure 10). The maximum protective effect was demonstrated by the “citrated” cerium sample (C1-2; 19.8 ± 0.4%), while the minimum was found for the “uncoated” cerium (C3-3; 5.7 ± 0.2%).

Thus, all of the uncoated nanocerium samples and the studied nanocerium composites demonstrated a statistically significant antioxidant effect. Using tocopherol as a control antioxidant yielded more accurate activity characteristics across groups than methyl viologen. However, the values differed only in absolute values, repeating themselves for the maximum and minimum antioxidant activity. As expected, the maximum antioxidant effects were obtained at the maximum concentration of the smallest nanocerium citrate composites. Uncoated nanocerium exhibited relatively weak activity against the superoxide anion radical. However, uncoated nanocerium tended to exhibit the greatest antioxidant activity against hydrogen peroxide, suggesting its effectiveness against the most damaging peroxide–lipid component causing oxidative stress.

### 2.5. Promutagenic and Antimutgenic Activity of CeO_2_NPs

Using the bioluminescent *E. coli* strain MG1655 (pRecA-lux), the complete absence of promutagenic effects due to DNA damage was demonstrated for all of the studied CeO_2_ nanoparticle samples (Table 4).

**Figure 11:**
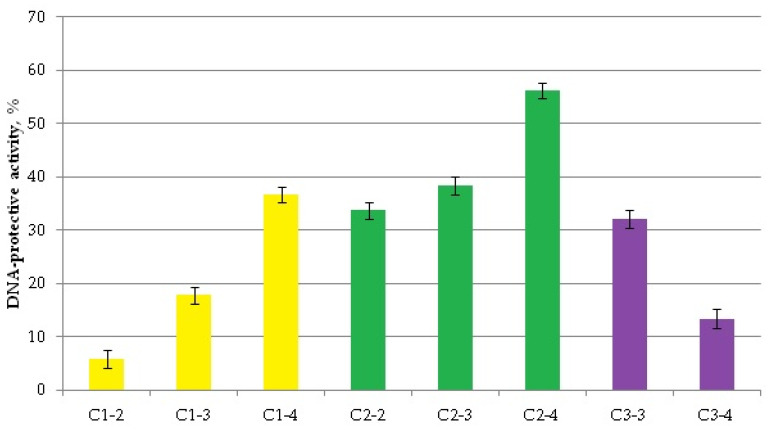
Antigenotoxic activity of CeO_2_NPs against the model toxicant dioxidine. All values significantly differed from zero (indicating no protective activity) (two-tailed Student’s *t*-test, Bonferroni-corrected *p* < 0.05).

The antimutagenic (antigenotoxic) activity of CeO_2_ nanoparticles ranged from 56.2 ± 1.3% for C2-4 to 5.8 ± 1.6% for C1-2 (Figure 11), with all values significantly differing from the control (*p* < 0.05, assessment based on initial luminescence values)

On average, “dextran” cerium exhibited stronger antimutagenic activity compared to CeO_2_ synthesized by other methods. However, there were no statistically significant intergroup differences.

## 3. Discussion

The toxicity of CeO_2_ particles against living organisms can be determined by a number of factors, including the concentration and size of the nanoparticles, their physicochemical properties, particle behavior in solutions, and their ability to agglomerate. In this study, we compared CeO_2_ nanoparticles synthesized using three different methods, each with a different particle size: up to 5 nm “dextran” nanocerium and “citrate” nanocerium, and 6–9 nm on average “uncoated” nanocerium [13,14,15,16,17]. Thus, the nanoparticle sizes were within the limits recommended by other researchers who have studied and confirmed the positive effects of nanocerium and its composites with no signs of toxicity [46]. An additional goal of this study was to determine the possible biological activity of nanoceria synthesized using several methods during storage for up to 12 months at room temperature.

Low toxicity. Overall, the bioassays using bacterial biosensors showed that, at the selected physiological concentrations, the CeO_2_NPs did not exert a significant, reliable toxic effect on the biosensor microbial strain cells. These data are consistent with the results obtained by other researchers when testing CeO_2_NPs in model organisms, which also demonstrated absent or insignificant toxicity. For example, Villa et al. [28] found no significant effect on the luminescence of the Gram-negative bacterium *Aliivibrio fischeri* using the Microtox^®^ bioassay; the percentage of inhibition was below 20% for bacteria exposed to all CeO_2_NPs. Uncoated and carbohydrate-coated CeO_2_ nanoparticles at a concentration of 200 mg × L^−1^ were not toxic to *Vibrio fischeri* NRRL B-11177, *Daphnia magna*, or *Danio rerio* [50].

### 3.1. Antibacterial Activity

Of all the samples studied, the cerium citrate nanoparticles at the highest concentration tested (10^−2^ M) most often demonstrated the ability to suppress the fluorescence and growth of biosensor bacterial strains. This is likely due to the direct adsorption of positively charged nanoparticles to the bacterial cell wall and thus replicates the results of other studies. Cheng et al. [51] showed that chitosan-coated CeO_2_ nanozymes not only exhibited good ROS scavenging capacity but also exhibited antibacterial activity. When evaluating the antibacterial activity of the CeO_2_–H_2_O_2_ system on the model bacterium *E. coli*, it was found that CeO_2_ alone at a concentration of 0.25 mg/mL induced a weak antibacterial effect [52]. CeO_2_NPs dispersed in an aqueous solution exhibited antibacterial activity against *Pseudomonas aeruginosa*, with minimum bactericidal concentrations for the ATCC reference strain and clinical isolate being 50 μg/mL and 200 μg/mL [53].

It is worth considering that the antibacterial effect of CeO_2_NPs may be stronger for Gram-negative *E. coli* than for Gram-positive strains. This may be due to the fact that Gram-positive bacteria have a thick outer peptidoglycan layer that contains linear polysaccharide chains with short peptides, which together form a rigid structure that is difficult for CeNPs to penetrate. In addition, a probable mechanism is the higher absorption of CeO_2_NPs on the cell wall [54]. Positively charged CeO_2_NPs can displace divalent cations that stabilize the lipopolysaccharide of the outer membrane, increasing its permeability [55]. The dependence of the growth inhibition rate of *E. coli* HB101 K-12 on the size and concentration of CeO_2_NPs was shown by Dar et al. [56] for four different-sized CeO_2_ samples. Weak to moderate antibacterial activity against various bacterial strains was shown by Butt et al. [57] for CeO_2_ nanoparticles obtained using an aqueous extract of *Cassia glauca*. In our previous work, the antimicrobial activity of citrate-stabilized CeO_2_NP solutions of varying concentrations was assessed against strains of *Bacillus subtilis*, *Bacillus cereus*, *Staphylococcus aureus*, *Pseudomonas aeruginosa*, *Escherichia coli*, *Proteus vulgaris*, *Candida albicans*, and *Aspergillus brasielensis* [58]. Nanocerium had a significant effect on the number of *E. coli*, exhibiting a bacteriostatic effect that was most pronounced at concentrations of 10^−2^–10^−3^ M.

### 3.2. Antioxidant Activity

All of the nanocerium samples studied herein exhibited good antioxidant activity against peroxide stress, and their effectiveness was not significantly different from those produced using the nanocerium synthesis method. The obtained data are in good agreement with the results of other studies. For example, it has been shown that CeO_2_ nanoparticles reduced oxidative damage caused by H_2_O_2_ in primary osteoblasts [2], bone marrow stem cells [32], retinal photoreceptors, liver diseases [59], and cardiomyopathy [60].

Moreover, the nanoparticles were more effective against oxidative stress caused by hydrogen peroxide than that caused by a superoxide anion radical inducer. Thus, for the “citrate” cerium sample C1-2, the protective activity against peroxide was 65.6%, while that against superoxide was only 19.8%.

The catalase-mimetic activity of the CeO_2_ nanoparticles we studied (inactivation of H_2_O_2_) is likely due to their lower Ce^3+^ content and, consequently, the higher Ce^4+^ surface content. It has been shown that a high Ce^3+^/Ce^4+^ ratio of surface Ce atoms imparts superoxide dismutase (SOD) mimetic activity, while a lower Ce^3+^/Ce^4+^ ratio leads to catalase mimetic activity [61]. This is consistent with the relatively low antioxidant activity of the CeO_2_ nanoparticles synthesized in this study against the superoxide anion radical, i.e., their low activity as an SOD mimetic.

In addition to the Ce^3+^/Ce^4+^ ratio, the insignificant protective properties of the studied CeO_2_ nanoparticles against the superoxide anion radical may likely be due to interaction with the phosphate buffer in which the samples were diluted. Singh et al. [62] investigated the change in the activity of nanozymes upon their dispersal in biologically relevant buffers and found that CeO_2_NPs lost their SOD-like activity when dispersed in a phosphate buffer (pH 7.4).

### 3.3. Lack of Prooxidant and Genotoxic Properties at Biocompatible Concentrations

In this study, the lack of prooxidant properties displayed by the CeO_2_ nanoparticles can be explained by their crystal lattice structure, as CeO_2_NPs have been shown to be prooxidants rather than antioxidants depending on their aggregation state, and chronic exposure to aggregates has been associated with elevated ROS levels by other researchers [25]. Suspending the nanoparticles in phosphate buffer also contributes to a decrease in the prooxidant activity of CeO_2_ due to the affinity of surface Ce^3+^ for phosphate anions, which occupy surface oxygen vacancies, forming CePO_4_. This compound modifies the nanocerium surface and blocks the redox cycle between Ce^3+^ and Ce^4+^. Increasing the concentration of cerium citrate nanoparticles resulted in enhanced antioxidant properties against peroxide, but also in toxicity (albeit not exceeding the level of the model toxicant). Pulido-Reyes et al. (2015) [38] showed that the percentage of surface Ce^3+^ correlated with toxicity and was the main factor explaining the observed toxic effect of nanocerium on the green algae *Pseudokirchneriella subcapitata*.

The complete absence of a genotoxic effect, determined using the *E. coli* biosensor strain MG1655 (pRecA-lux), may indicate that the nanoparticles synthesized in this study do not penetrate the cell and do not mediate indirect genotoxicity. This mechanism involves CeO_2_NPs adsorbing to the cell surface, inactivating proteins, and thus entering the cell, where they inactivate cellular enzymes. This leads to the release of hydrogen peroxide, damaging vital structures such as DNA, RNA, and proteins.

### 3.4. Antigenotoxic (DNA-Protective) Effects

Simultaneously, we discovered that CeO_2_NPs exhibit antimutagenic properties against the prooxidant mutagen 1,4-dioxide 2,3-quinoxaline dimethanol (dioxidine). Thus, we can assume that the mechanism of CeO_2_NPs’ antimutagenic action is similar to that of their antioxidant action against hydrogen peroxide, i.e., CeO_2_NPs protect DNA from damage caused by ROS. The “dextran” CeO_2_NPs, which were the smallest, on average exhibited slightly higher antimutagenic activity against dioxidine compared to other samples. Moreover, the level of protection increased as the nanoparticle concentration decreased (33.7–56.2%). Thus, bioassays using bacterial biosensors demonstrated the low toxicity of the studied CeO_2_NPs, their antioxidant activity against hydrogen peroxide, and their DNA-protective activity against oxidative damage. No prooxidant or genotoxic properties were detected for CeO_2_NPs.

Thus, the absence of significant differences in biosensor performance when co-cultured in differently synthesized nanocerium particles allows for the selection of the simplest and most technologically advanced method for substrate synthesis to create a biomedical product.

#### Limitations and Prospects

The authors recognize that all research studies have limitations, and that the emergence of new answers simultaneously creates new questions, which in turn create future research avenues. This research was carried out over 6–12 months following the synthesis and physicochemical characterization of the nanoparticles. We did not conduct an exhaustive full-fledged dynamic monitoring of the physical and chemical state of the nanoparticles and their solutions during this time, based on the assumption that, in such a relatively short period, at a relatively stable temperature of around 21 °C and without exposure to chemical or physical agents, the physical and chemical characteristics would remain stable. However, the authors understand that to use the substances under study as pharmaceuticals, it would be very useful to study the effects of parameters such as time, pH, temperature, storage conditions, and the Ce^3+^/Ce^4+^ ratio in order to understand the storage of the final drug product, its colloidal stability, and its properties related to redox processes. We wish to underscore that the main purpose of this study was to investigate the biological effects of CeO_2_NPs synthesized previously by different methods, using a set of different bacterial biosensors to determine their overall toxicity, pro-/antioxidant status, and pro-/antimutagenic properties. Our future research will focus on the nanoceria stability during long-term storage and the creation of biomedical products.

Limitations of the bacterial biosensor bioassay method include the lack of a means for CeO_2_ to penetrate bacterial cells with a sufficiently dense cell wall (unlike, for example, animal cells), which precludes assessing potential damage to intracellular components. On the other hand, during the preliminary screening of various synthesized particles of this class, this method can immediately eliminate samples with obvious toxicity, allowing for further, more detailed study of samples that show no toxic effects.

The results of the bioassay using cerium dioxide nanoparticles provide optimism for further study to assess the potential clinical application of cerium nanocomposites in human and veterinary medicine. The demonstrated biocompatibility of CeO_2_NPs, combined with their pronounced antioxidant and antigenotoxic activity, provides the basis for the development of innovative therapeutic approaches for the treatment of diseases associated with oxidative stress. Promising applications include neuroprotection in stroke and traumatic brain injury, treatment of chronic wounds and diabetic ulcers, treatment of inflammatory liver diseases, and ophthalmic treatment of retinal degenerative diseases.

The unique ability of CeO_2_NPs to mimic the activity of endogenous antioxidant enzymes (superoxide dismutase, catalase) while maintaining their regenerative properties makes them ideal candidates for the development of long-term therapeutic systems. Further development in this area can be aimed at creating functionalized nanoparticles with improved bioavailability, targeted delivery, and controlled release of active compounds, which could revolutionize treatment approaches for a wide range of pathologies.

## 4. Materials and Methods

### 4.1. Nanocerium Synthesis

#### 4.1.1. Citric Nanocerium, C-1

Solutions of cerium(III) nitrate and citric acid (50 mL) were mixed, with the Ce(NO_3_)_3_·6H_2_O:citric acid molar ratio being 1:1. To the continuously mixed 3 M aqueous ammonia, a solution of cerium(III) nitrate and citric acid was added rapidly. The final mixture was stirred for 12 h. To the solution obtained, an excess of isopropanol was added, and the mixture was refluxed to form a white precipitate. The precipitate was further washed with isopropanol several times and dried in air at 60 °C. Nanoceria solution was prepared by dispersing the powder in distilled water. The nanoparticles obtained by this method were predominantly 3–4 nm in size, according to the diameter of the inorganic CeO_2_ core [14,15].

#### 4.1.2. Dextran Nanocerium, C-2

A mixed solution with a total volume of 50 mL (prepared with distilled water) was prepared that contained cerium(III) nitrate hexahydrate (Ce(NO_3_)_3_·6H_2_O, 99.99%, LANHIT, Russia, Moscow; molecular weight 434.23 326.13 anhydrous) and dextran (Mr = 6000 Da, abcr GmbH, Karlsruhe, Germany) at a mass ratio of cerium(III) nitrate hexahydrate to dextran of 1:2 (Ce2D). To the continuously stirred solution, 3 M aqueous ammonia (analytical-grade, Khimmed, Russia, Moscow) was added dropwise over a period of 3 h, maintaining the pH at 7.5–8.0. After the pH stabilized, the mixture was stirred for a further 2 h. Then, aqueous ammonia was added until the pH reached 12, followed by additional stirring for 24 h. The nanoparticles obtained by this method were predominantly 1–2 nm in size, according to the diameter of the inorganic CeO_2_ core [16].

#### 4.1.3. Uncoated Nanocerium, C-3

The cerium dioxide nanoparticle powder (C-3) [13] was synthesized through the thermal decomposition of cerium carbonate obtained by precipitation from a nitrate aqueous solution, according to the following reaction equations:

2CeO_2_ + 6HNO_3_ +H_2_O_2_ → 2Ce(NO_3_)_3_ + O_2_↑ + 4H_2_O (1)

2Ce(NO_3_)_3_ + 3(NH_4_)_2_CO_3_ → Ce_2_(CO_3_)_3_ + 6NH_4_NO_3_ (2)

Ce_2_(CO_3_)_3_ → 2CeO_2_ + 3CO_2_ (3)

The initial synthesis of the starting cerium nitrate solution was carried out using calcined cerium oxide with a purity of 99.99% (LANHIT, Moscow, Russia) and nitric acid grade “OSCh 18-4” (70–70.2 wt%, SigmaTek LLC, Moscow, Russia). Immediately after precipitation, the resulting cerium(III) carbonate precipitate was filtered and washed with double-distilled water at room temperature until a negative test for nitrate ions was achieved. The precipitate was air-dried at 20 °C; then, the dried cerium(III) carbonate was transferred to a quartz cuvette and calcined in a muffle furnace at 450 °C for two hours. Next, cerium dioxide powder was dissolved in distilled water to form a 0.1 M suspension, which was dispersed and sedimented for 72 h to obtain finely dispersed particles in the upper third of the fraction. According to transmission electron microscopy and X-ray diffraction analysis, the nanoparticle size did not exceed 10 nm, with a typical range of 6–9 nm. This synthesis technology has proven to be optimal under production conditions, ensuring the best tissue regenerative activity and biocompatibility [13].

### 4.2. Cerium Oxide Nanoparticle Characterization

In this study, we used three types of nanoceria, synthesized according to the protocols detailed in our previous research [13,14,15,16].

The structure and size of cerium oxide nanoparticles was studied via transmission electron microscopy (TEM) using a JEM 2100 (JEOL, Akishima, Japan) microscope at the maximum accelerating voltage of 200 kV. TEM studies were performed at the National University of Science and Technology MISIS (Moscow, Russia). TEM allowed us to determine the morphology of nanoparticles without their shell (coating).

The particle sizes with shells and ζ-potential were recorded using the dynamic light scattering method (DLS) on a Zetasizer Nano ZS (Malvern Instruments, Malvern, UK).

X-ray diffraction images of the powders were obtained at room temperature using a Rigaku Ultima IV CoKα X-ray diffractometer at diffraction angles Θ from 10 to 120°.

The present biomedical study was performed on nanoceria samples synthesized 12 months before the start of the study; all three samples were stored at room temperature. Re-examining the nanoceria samples using TEM with an electron diffraction pattern (Figure 1 and Figure 2), DLS, and ζ-potential (Figure 3) gave us the opportunity to compare the results obtained with those from 12 months ago [13,14,15,16] to confirm the stability of the results and to estimate the storage life of the nanoscale samples, which is crucial for drug creation.

Before the present study, the nanocerium samples were dispersed in bidistilled water to the required concentration and treated in a UZ-bath. Sample concentrations were studied using inductively coupled plasma-mass spectrometry (Elan DRC-e, Perkin Elmer, Madrid, Spain).

This work is a continuation of consistent research devoted to the development of a new type of regenerative drug based on redox-active cerium-containing nanomaterials. In our previous works, we synthesized and studied nanocerium obtained via different synthesis techniques, as well as to evaluate the influence of various CeO_2_ concentrations on cells involved in skin structure regeneration (human mesenchymal stem cells, keratinocytes, and fibroblasts) in order to select the most optimal nanoceria variants with the best biomedical [13,14,15,16,17]. These studies also determined the non-cytotoxicity of CeO_2_NPs in a wide range of concentrations (10^−2^–10^−6^ M = 0.000001–0.01 mol/L) and established the most optimal range of nanoceria concentrations for further development of the regenerative agent. In this work, the best CeO_2_NPs and their best concentrations were used.

#### 4.2.1. Bacterial Strains

##### Bioluminescent *E. coli* Strain

Bioluminescent *E. coli* MG1655 strains carry plasmids with *lux-CDABE* reporter genes placed under the control of genotoxic and oxidative stress promoters and the ampicillin resistance gene *bla*. The *E. coli* MG1655 pXen7-lux strain carries a constitutive, constantly expressed promoter and is used to detect general toxicity or the effect of test substances on the bioluminescence process itself. The latter eliminates false-positive results when testing on inducible biosensors of the same series. The *E. coli* MG1655 pRecA-lux strain with the SOS-inducible PrecA promoter detects the penetration of DNA-damaging agents into cells; the *E. coli* MG1655 pKatG-lux and *E. coli* MG1655 pSoxS-lux strains, with promoters inducible by peroxides and superoxide, respectively, detect the occurrence of oxidative stress [40].

The RecA protein catalyzes homologous pairing and strand exchange of DNA molecules necessary for DNA recombinational repair. The RecA promoter is partially active in the absence of stress, and when DNA defects that block replication, such as single-strand breaks and pyrimidine dimers, occur, the SOS regulon repressor LexA is inactivated, and also, RecA transcription directly correlates with the amount of DNA damage [63].

KatG is an enzyme with catalase and peroxidase activity [64]; its transcription is enhanced upon the penetration of peroxides into the cell, and this directly correlates with the intracellular concentration of hydrogen peroxide [40].

SoxS is a transcriptional activator of the superoxide response regulon, which comprises at least 10 genes, including the superoxide dismutase gene *sodA* [65]. Transcription at this promoter is induced by an increase in the concentration of superoxide anions and nitric oxide in the cell [40].

The *E. coli* XL1-Blue pNK3751 bioluminescent strain expresses the genes encoding the enzymes comprising the bacterial autonomous bioluminescence system [66]. The iLux operon is under the control of the tac promoter, which ensures high levels of expression. The iLux vector (ilux pGEX(-)) (pNK3751) used in this study was purchased from Addgene (Plasmid #107879, Watertown, MA, USA). Colony selection was performed using the selective antibiotic ampicillin.

##### Fluorescent *E. coli* Strains

*E. coli* XL1-Blue eGFP and *E. coli* XL1-Blue FusionRed are *E. coli* XL1-Blue bacterial strains expressing the green fluorescent protein eGFP gene (Ex 488 nm, Em 507 nm) [67] and red fluorescent protein FusionRed gene (Ex 580 nm, Em 608 nm) [68], respectively. The strains were obtained by transforming bacteria with the pQE-30 expression vector (Qiagen, Venlo, The Netherlands), into which the fluorescent protein genes were cloned at the BamHI and HindIII restriction sites. Colony selection was carried out using the selective antibiotic ampicillin. Expression of fluorescent proteins was under the control of the T5 promoter, which has a basal level of expression and synthesis of fluorescent protein in the cell occurs continuously, without induction.

#### 4.2.2. Cultivation Conditions

Bioluminescent strains of *E. coli* MG1655 and fluorescent strains of *E. coli* XL1-Blue were inoculated in 5 mL of lysogeny broth (LB, Miller’s formulation) (10 g/L peptone, 10 g/L NaCl, 5 g/L yeast extract) with the addition of ampicillin 125 μg/mL [69] and incubated in a Biosan ES-20 thermoshaker at 37 °C and 200 rpm. The next day, 5 mL of fresh LB with ampicillin was added to the overnight culture, then incubated at 37 °C for 1–1.5 h and 0.5 h before the experiment, and diluted to a density of 0.05 McFarland units; culture turbidity was measured using a DEN-1B densitometer (Biosan, Riga, Latvia).

#### 4.2.3. Assessment of CeO_2_NP Overall Toxicity Using Bacterial Biosensors

To determine the toxicity of the studied nanoparticles, the constitutively luminescent fluorescent strains *E. coli* XL1-BLue eGFP and *E. coli* XL1-Blue FusionRed and the bioluminescent strains *E. coli* XL1-BLue pNK3751 and *E. coli* MG1655 pXen7-lux were used. The toxicity of the nanoparticles was assessed based on the degree of decrease in fluorescence/luminescence intensity and the growth of bacterial strains in the presence of nanoparticle solutions. If a decrease in culture turbidity (OD600) was observed while the luminescence level remained unchanged, the effect was considered bacteriostatic.

A volume of 180 μL of the overnight culture of the biosensor strains, prepared as described in Section 4.2.2. Cultivation Conditions, and 20 μL of the Ce nanoparticle solution were added to the wells of a Greiner 96 Flat-Bottom 96-well microplate (Greiner Bio-One, Kremsmünster, Austria). For the positive controls, 20 μL of the model toxicant (ZnSO_4_) solution at a final concentration of 0.1 g/L was added to the wells instead of nanoparticles. To monitor the dose dependence of fluorescence suppression on the bacteriostatic concentration, ZnSO_4_ solution was added at concentrations 2 and 3 times higher than the working concentration (0.2 and 0.3 g/L). A volume of 20 μL of phosphate buffer (solvent control) was added to the wells of the negative control. The microplate was placed in a FLUOstar Omega multimode plate reader (BMG Labtech, Ortenberg, Germany) with a thermostat and incubated at 37 °C and 200 rpm (double orbital). A blank for measuring the fluorescence and optical density (OD600) of the culture (160 μL LB with ampicillin and 40 μL distilled water) was used. To determine the effect of nanoparticle concentrations on *E. coli* growth curves, the optical density of the cultures was measured every 10 min at a wavelength of 600 nm and temperature of 37 ± 0.2 °C using a FLUOstar Omega instrument before measuring the fluorescence/luminescence. Bioluminescence and fluorescence intensities were also measured every 10 min. To measure the fluorescence of *E. coli* XL1-Blue eGFP, FLUOstar Omega fluorescent (λex/λem) 485–12/520–10 filters were used; to measure the fluorescence of *E. coli* XL1-Blue FusionRed, 544–10/590–10 filters were used. Since ZnSO_4_ and the tested nanoparticles also fluoresce at the above-mentioned wavelengths, to calculate the intrinsic fluorescence of the *E. coli* XL1-BlueeGFP and *E. coli* XL1-Blue FusionRed strains, the fluorescence values with the culture and the substance were subtracted from the fluorescence values with the nutrient medium and the substance.

For constitutive strains, the suppression of luminescence (*S^L^*) was calculated using the following formula:

(4) $$S^{L}=\left(1-\frac{L_{e}}{L_{c}}\right)\times 100$$

where L_e_ is the luminescence/fluorescence intensity of the sample containing the test substance (in relative light units, RLU), and L_c_ is the luminescence/fluorescence intensity of the control sample (in RLU).

Since the tested nanoparticles affect the turbidity of the culture, to calculate the intrinsic turbidity of the constitutive strains, the luminescence values for the culture medium and the corresponding nanoparticle solutions were subtracted from the turbidity values for the culture and nanoparticle solutions.

Growth inhibition $(S^{G})\;$ was calculated using the following formula:

(5) $$S^{G}=\left(1-\frac{{OD}_{600}^{e}}{{OD}_{600}^{c}}\right)\times 100$$

where ${OD}_{600}^{e}$—optical density of a sample with a testing substance at 600 nm; ${OD}_{600}^{c}$—optical density of control sample.

Conclusions regarding toxicity were drawn by comparing nanoparticle-induced growth inhibition values (measured by OD_600_) with MIC_50_ (the minimum inhibitory concentration required to inhibit 50% of strain growth), as well as by assessing the complete fluorescence or luminescence inhibition concentration of the biosensors [70,71].

#### 4.2.4. Pro-/Antioxidant and DNA-Disruptive/-Protective Activity Assessment of CeO_2_NPs

The genotoxic and prooxidant properties of cerium nanoparticles, as well as their protective properties, were studied using the inducible *E. coli* strains MG1655 (pRecA-lux), MG1655 (pKatG-lux), and MG1655 (pSoxS-lux).

The genotoxicity and prooxidant properties of the nanoparticles were determined by inducing fluorescence in the biosensor strains in their presence. For this purpose, 180 µL of an overnight *E. coli* MG1655 culture and 20 µL of the nanoparticle solution were added to the wells of a Greiner 96 Flat-Bottom 96-well microplate (Greiner Bio-One, Kremsmünster, Austria). Negative control wells were supplemented with 20 μL of phosphate buffer (solvent control) instead of nanoparticles.

To assess protective activity, 160 μL of an overnight *E. coli* MG1655 culture, 20 μL of nanoparticle solution, and 20 μL of inducers were added to the wells of the plate: oxidative stress inducers included hydrogen peroxide and methyl viologen at a final concentration of 400 μM in the plate; the genotoxic stress inducer was dioxidine (1,4-dioxide 2,3-quinoxaline dimethanol) at 9 μM. To monitor the dose dependence of the induction coefficient on inducer concentration, inducers were added at half the working concentration: hydrogen peroxide and methyl viologen at a concentration of 200 μM, and dioxidine at 4.5 μM.

The SOS response induction factor (I) was calculated using the following formula:

(6) $$I=\frac{L_{e}}{L_{c}}-1$$

where L_e_ is the luminescence intensity of the sample with the inducer (nanoparticles) (RLU); L_c_ is the luminescence intensity of the control sample (RLU).

Protective activity (P, %) was calculated using the following formula:

(7) $$P=\left(1-\frac{I_{e}}{I_{c}}\right)\times 100$$

where I_e_ and I_c_ are the luminescence induction coefficients in the presence and absence of nanoparticle solutions, respectively.

### 4.3. Statistical Analysis

Normality of data distribution was assessed using the Shapiro–Wilk test. For comparisons between each nanoparticle-treated group and the corresponding control, a two-tailed Student’s *t*-test for independent samples (parametric) was used. Equality of variances was assessed using Levene’s test, and if violated, Welch’s correction was applied. To control the family-wise error rate in the presence of multiple comparisons, *p*-values were adjusted using the Bonferroni correction.

All results are presented as the mean ± standard error of the mean (SEM; *n* ≥ 3 replicates per time point) with Bessel’s correction. Statistically significant differences (*p* < 0.05) are marked with asterisks (*).

To calculate the error of the average percentage of suppression and the induction coefficient, the error propagation formula for the ratio of two means was used, with subsequent conversion to percentages if necessary.

(8) $${SEM}_{inhibition}100\times \sqrt{{\left(\frac{{SEM}_{exp}}{\underline{exp}}\right)}^{2}+{\left(\frac{{SEM}_{ctrl}}{\underline{ctrl}}\right)}^{2}}$$

Standard error of the mean of protective activity (SEM_P_) was calculated using the following formula:

(9) $${SEM}_{P}=100\times \sqrt{{\left(\frac{100}{I_{c}}\right)}^{2}\times {SEM}_{I_{c}}^{2}+{\left(\frac{100\times I_{e}}{I_{c}^{2}}\right)}^{2}\times {SEM}_{I_{c}}^{2}}$$

where I_e_—mean experimental induction value; I_c_—mean control induction value SEM I_e_; and SEM I_c_—corresponding standard mean errors.

## 5. Conclusions

CeO_2_ nanoparticles synthesized using three different methods demonstrated comparable biological effects. All were nontoxic and, therefore, biocompatible. They demonstrated good antioxidant properties against hydrogen peroxide (especially “citrate” cerium) and moderate activity against the superoxide anion radical. High DNA-protective activity was also noted. At the tested concentrations, the CeO_2_NPs demonstrated the complete absence of prooxidant and genotoxic effects. Together, these findings confirm the need for further research in this area and suggest significant potential for the development of pharmaceuticals using nanocerium composites for human and veterinary medicine.

## Figures and Tables

**Table 1:** Tested nanoparticles and their concentrations in the samples.

No.	Short n.	Substance	Concentration	Nanoparticle Sizes, nm (According to TEM Data)	Synthesis and Characterization
1	C1-2	Citric nanocerium	10^−2^ M	<5	[14,15,17]
2	C1-3		10^−3^ M	<5	
3	C1-4		10^−4^ M	<5	
4	C2-2	Dextran nanocerium	10^−2^ M	<5	[16,17]
5	C2-3		10^−3^ M	<5	
6	C2-4		10^−4^ M	<5	
7	C3-3	Uncoated nanocerium	10^−3^ M	6–9	[13]
8	C3-4		10^−4^ M	6–9	

**Table 2:** Maximum luminescence induction factors for the *E. coli* strain MG1655 (pKatG-lux) in the presence of CeO_2_NPs. *—*p* < 0.05 (Student’s *t*-test).

Sample	H_2_O_2_	C 1-2	C 1-3	C 1-4	C 2-2	C 2-3	C 2-4	C 3-3	C 3-4
Maximal induction factor	17.2 ± 0.11 *	0.03 ± 0.07	0.3 ± 0.03	0.5 ± 0.09	0.5 ± 0.11	0.6 ± 0.05	0.7 ± 0.02	0.8 ± 0.01	0.3 ± 0.04

**Table 3:** Maximum luminescence induction factors for *E. coli* strain MG1655 (pSoxS-lux) in the presence of CeO_2_NPs.

Sample	Methyl Viologen	C 1-2	C 1-3	C 1-4	C 2-2	C 2-3	C 2-4	C 3-3	C 3-4
Maximal induction factor	13.8 ± 0.11	0.05 ± 0.02	0.03 ± 0.01	0.03 ± 0.02	0.06 ± 0.01	0.03 ± 0.01	0.01 ± 0.007	0.04 ± 0.005	0.01 ± 0.007

**Table 4:** Maximum luminescence induction coefficients of the *E. coli* strain MG1655 (pRecA-lux) in the presence of CeO_2_NPs.

Sample	Dioxidine	C 1-2	C 1-3	C 1-4	C 2-2	C 2-3	C 2-4	C 3-3	C 3-4
Max induction factor I	15.0 ± 0.05	0.07 ± 0.005	0.09 ± 0.03	0.3 ± 0.002	0.3 ± 0.003	0.5 ± 0.01	0.7 ± 0.05	0.4 ± 0.005	0.2 ± 0.005

## Data Availability

Data available on request due to restrictions. The data presented in this study are available on request from the corresponding author.

## References

[1] Mousaiyan S., Baharara J., Es-haghi A. (2024). Biopreparation of Cerium Oxide Nanoparticles Using Alginate: Characterization and Estimation of Antioxidant and Its Activity against Breast Cancer Cell Lines (MCF7). Results Chem..

[2] Jairam L.S., Chandrashekar A., Prabhu T.N., Kotha S.B., Girish M.S., Devraj I.M., Dhanya Shri M., Prashantha K. (2023). A Review on Biomedical and Dental Applications of Cerium Oxide Nanoparticles—Unearthing the Potential of This Rare Earth Metal. J. Rare Earths.

[3] Luo M., Shaitan K., Qu X., Bonartsev A.P., Lei B. (2022). Bioactive Rare Earth-Based Inorganic-Organic Hybrid Biomaterials for Wound Healing and Repair. Appl. Mater. Today.

[4] Casals E., Zeng M., Parra-Robert M., Fernández-Varo G., Morales-Ruiz M., Jiménez W., Puntes V., Casals G. (2020). Cerium Oxide Nanoparticles: Advances in Biodistribution, Toxicity, and Preclinical Exploration. Small.

[5] Dutta D., Mukherjee R., Patra M., Banik M., Dasgupta R., Mukherjee M., Basu T. (2016). Green Synthesized Cerium Oxide Nanoparticle: A Prospective Drug against Oxidative Harm. Colloids Surf. B Biointerfaces.

[6] Akanchise T., Angelova A. (2023). Potential of Nano-Antioxidants and Nanomedicine for Recovery from Neurological Disorders Linked to Long COVID Syndrome. Antioxidants.

[7] Singh S. (2024). Antioxidant Nanozymes as Next-Generation Therapeutics to Free Radical-Mediated Inflammatory Diseases: A Comprehensive Review. Int. J. Biol. Macromol..

[8] Chai W.F., Tang K.S. (2021). Protective Potential of Cerium Oxide Nanoparticles in Diabetes Mellitus. J. Trace Elem. Med. Biol..

[9] Raut S.K., Khullar M. (2023). Oxidative Stress in Metabolic Diseases: Current Scenario and Therapeutic Relevance. Mol. Cell. Biochem..

[10] Hajam Y.A., Rani R., Ganie S.Y., Sheikh T.A., Javaid D., Qadri S.S., Pramodh S., Alsulimani A., Alkhanani M.F., Harakeh S. (2022). Oxidative Stress in Human Pathology and Aging: Molecular Mechanisms and Perspectives. Cells.

[11] Chaudhary M.R., Chaudhary S., Sharma Y., Singh T.A., Mishra A.K., Sharma S., Mehdi M.M. (2023). Aging, Oxidative Stress and Degenerative Diseases: Mechanisms, Complications and Emerging Therapeutic Strategies. Biogerontology.

[12] Kardan T., Mohammadi R., Taghavifar S., Cheraghi M., Yahoo A., Mohammadnejad K. (2021). Polyethylene Glycol-Based Nanocerium Improves Healing Responses in Excisional and Incisional Wound Models in Rats. Int. J. Low. Extrem. Wounds.

[13] Silina E.V., Stupin V.A., Manturova N.E., Chuvilina E.L., Gasanov A.A., Ostrovskaya A.A., Andreeva O.I., Tabachkova N.Y., Abakumov M.A., Nikitin A.A. (2024). Development of Technology for the Synthesis of Nanocrystalline Cerium Oxide Under Production Conditions with the Best Regenerative Activity and Biocompatibility for Further Creation of Wound-Healing Agents. Pharmaceutics.

[14] Silina E.V., Ivanova O.S., Manturova N.E., Medvedeva O.A., Shevchenko A.V., Vorsina E.S., Achar R.R., Parfenov V.A., Stupin V.A. (2024). Antimicrobial Activity of Citrate-Coated Cerium Oxide Nanoparticles. Nanomaterials.

[15] Silina E.V., Stupin V.A., Manturova N.E., Ivanova O.S., Popov A.L., Mysina E.A., Artyushkova E.B., Kryukov A.A., Dodonova S.A., Kruglova M.P. (2023). Influence of the Synthesis Scheme of Nanocrystalline Cerium Oxide and Its Concentration on the Biological Activity of Cells Providing Wound Regeneration. Int. J. Mol. Sci..

[16] Silina E.V., Manturova N.E., Ivanova O.S., Baranchikov A.E., Artyushkova E.B., Medvedeva O.A., Kryukov A.A., Dodonova S.A., Gladchenko M.P., Vorsina E.S. (2024). Cerium Dioxide–Dextran Nanocomposites in the Development of a Medical Product for Wound Healing: Physical, Chemical and Biomedical Characteristics. Molecules.

[17] Silina E.V., Manturova N.E., Sevastianov V.I., Perova N.V., Gladchenko M.P., Kryukov A.A., Ivanov A.V., Dudka V.T., Prazdnova E.V., Emelyantsev S.A. (2025). Development of a Collagen–Cerium Oxide Nanohydrogel for Wound Healing: In Vitro and In Vivo Evaluation. Biomedicines.

[18] Ma X., Cheng Y., Jian H., Feng Y., Chang Y., Zheng R., Wu X., Wang L., Li X., Zhang H. (2019). Hollow, Rough, and Nitric Oxide-Releasing Cerium Oxide Nanoparticles for Promoting Multiple Stages of Wound Healing. Adv. Healthc. Mater..

[19] Xue Y., Yang F., Wu L., Xia D., Liu Y. (2024). CeO_2_ Nanoparticles to Promote Wound Healing: A Systematic Review. Adv. Healthc. Mater..

[20] Huang Y., Zhang M., Jin M., Ma T., Guo J., Zhai X., Du Y. (2023). Recent Advances on Cerium Oxide-Based Biomaterials: Toward the Next Generation of Intelligent Theranostics Platforms. Adv. Healthc. Mater..

[21] Casals G., Perramón M., Casals E., Portolés I., Fernández-Varo G., Morales-Ruiz M., Puntes V., Jiménez W. (2021). Cerium Oxide Nanoparticles: A New Therapeutic Tool in Liver Diseases. Antioxidants.

[22] Tang J.L.Y., Moonshi S.S., Ta H.T. (2023). Nanoceria: An Innovative Strategy for Cancer Treatment. Cell. Mol. Life Sci..

[23] Jansson P.J., Kalinowski D.S., Lane D.J.R., Kovacevic Z., Seebacher N.A., Fouani L., Sahni S., Merlot A.M., Richardson D.R. (2015). The Renaissance of Polypharmacology in the Development of Anti-Cancer Therapeutics: Inhibition of the “Triad of Death” in Cancer by Di-2-Pyridylketone Thiosemicarbazones. Pharmacol. Res..

[24] Feng N., Liu Y., Dai X., Wang Y., Guo Q., Li Q. (2022). Advanced Applications of Cerium Oxide Based Nanozymes in Cancer. RSC Adv..

[25] Lord M.S., Berret J.F., Singh S., Vinu A., Karakoti A.S. (2021). Redox Active Cerium Oxide Nanoparticles: Current Status and Burning Issues. Small.

[26] Soltani M., Soltani M., Karami-Mohajeri S., Mohadesi A., Ranjbar M., Oghabian Z., Mehrpour O., Khosravi F. (2025). An Interdisciplinary Approach to Assessing the Toxicity Reduction of Cerium Oxide Nanoparticles Coated with Polyethylene Glycol and Polyvinylpyrrolidone Polymers: An in Vitro Study. Toxicol. Vitr..

[27] Arndt D.A., Oostveen E.K., Triplett J., Butterfield D.A., Tsyusko O.V., Collin B., Starnes D.L., Cai J., Klein J.B., Nass R. (2017). The Role of Charge in the Toxicity of Polymer-Coated Cerium Oxide Nanomaterials to *Caenorhabditis elegans*. Comp. Biochem. Physiol. Part C Toxicol. Pharmacol..

[28] Villa S., Maggioni D., Hamza H., Di Nica V., Magni S., Morosetti B., Parenti C.C., Finizio A., Binelli A., Della Torre C. (2020). Natural Molecule Coatings Modify the Fate of Cerium Dioxide Nanoparticles in Water and Their Ecotoxicity to Daphnia Magna. Environ. Pollut..

[29] Liu Y., Liao Y.-L., Wang J.-X., Zhang B.-F., Tang Q.-P., Ding Y., Zuo S.-J., Zhou Q.-H., Pei D.-S. (2025). The Effect and Mechanisms of Nano Cerium Dioxide (Nano-CeO_2_) on Cardiovascular Development in Zebrafish (*Danio rerio*). Environ. Pollut..

[30] Liman R., Acikbas Y., Ciğerci İ.H. (2019). Cytotoxicity and Genotoxicity of Cerium Oxide Micro and Nanoparticles by Allium and Comet Tests. Ecotoxicol. Environ. Saf..

[31] Benameur L., Auffan M., Cassien M., Liu W., Culcasi M., Rahmouni H., Stocker P., Tassistro V., Bottero J.-Y., Rose J. (2015). DNA Damage and Oxidative Stress Induced by CeO_2_ Nanoparticles in Human Dermal Fibroblasts: Evidence of a Clastogenic Effect as a Mechanism of Genotoxicity. Nanotoxicology.

[32] Hanafy B.I., Cave G.W.V., Barnett Y., Pierscionek B. (2020). Treatment of Human Lens Epithelium with High Levels of Nanoceria Leads to Reactive Oxygen Species Mediated Apoptosis. Molecules.

[33] Koehlé-Divo V., Cossu-Leguille C., Pain-Devin S., Simonin C., Bertrand C., Sohm B., Mouneyrac C., Devin S., Giambérini L. (2018). Genotoxicity and Physiological Effects of CeO_2_ NPs on a Freshwater Bivalve (*Corbicula fluminea*). Aquat. Toxicol..

[34] Farias I.A.P., Santos C.C.L., Xavier A.L., Batista T.M., Nascimento Y.M., Nunes J.M.F.F., Silva P.M.F., Menezes-Júnior R.A., Ferreira J.M., Lima E.O. (2021). Synthesis, Physicochemical Characterization, Antifungal Activity and Toxicological Features of Cerium Oxide Nanoparticles. Arab. J. Chem..

[35] Moro Druzian D., Rodrigues Oviedo L., Nunes Loureiro S., Dias Wouters R., Stefanello Vizzotto B., De Oliveira Pinto E., Julia Schűssler De Vanconcellos N., Patricia Moreno Ruiz Y., Galembeck A., Pavoski G. (2023). Cerium Oxide Nanoparticles: Biosynthesis, Characterization, Antimicrobial, Ecotoxicity and Photocatalytic Activity. J. Photochem. Photobiol. A Chem..

[36] Jung S.-K., Qu X., Aleman-Meza B., Wang T., Riepe C., Liu Z., Li Q., Zhong W. (2015). Multi-Endpoint, High-Throughput Study of Nanomaterial Toxicity in *Caenorhabditis elegans*. Environ. Sci. Technol..

[37] Savić-Zdravković D., Milošević D., Conić J., Marković K., Ščančar J., Miliša M., Jovanović B. (2021). Revealing the Effects of Cerium Dioxide Nanoparticles through the Analysis of Morphological Changes in *Chironomus riparius*. Sci. Total Environ..

[38] Pulido-Reyes G., Rodea-Palomares I., Das S., Sakthivel T.S., Leganes F., Rosal R., Seal S., Fernández-Piñas F. (2015). Untangling the Biological Effects of Cerium Oxide Nanoparticles: The Role of Surface Valence States. Sci. Rep..

[39] Lahive E., Jurkschat K., Shaw B.J., Handy R.D., Spurgeon D.J., Svendsen C. (2014). Toxicity of Cerium Oxide Nanoparticles to the Earthworm Eisenia Fetida: Subtle Effects. Environ. Chem..

[40] Bazhenov S.V., Novoyatlova U.S., Scheglova E.S., Prazdnova E.V., Mazanko M.S., Kessenikh A.G., Kononchuk O.V., Gnuchikh E.Y., Liu Y., Al Ebrahim R. (2023). Bacterial Lux-Biosensors: Constructing, Applications, and Prospects. Biosens. Bioelectron. X.

[41] Liu M., Yang W., Zhu W., Yu D. (2025). Innovative Applications and Research Advances of Bacterial Biosensors in Medicine. Front. Microbiol..

[42] Belkin S. (2003). Microbial Whole-Cell Sensing Systems of Environmental Pollutants. Curr. Opin. Microbiol..

[43] Chen Y., Guo Y., Liu Y., Xiang Y., Liu G., Zhang Q., Yin Y., Cai Y., Jiang G. (2023). Advances in Bacterial Whole-Cell Biosensors for the Detection of Bioavailable Mercury: A Review. Sci. Total Environ..

[44] Chistyakov V.A., Semenyuk Y.P., Morozov P.G., Prazdnova E.V., Chmykhalo V.K., Kharchenko E.Y., Kletskii M.E., Borodkin G.S., Lisovin A.V., Burov O.N. (2015). Synthesis and Biological Properties of Nitrobenzoxadiazole Derivatives as Potential Nitrogen(II) Oxide Donors: SOX Induction, Toxicity, Genotoxicity, and DNA Protective Activity in Experiments Using Escherichia Coli-Based Lux Biosensors. Russ. Chem. Bull..

[45] Emelyantsev S., Prazdnova E., Chistyakov V. (2025). Solubilizer of Bacterial Origin Surfactin Increases the Biological Activity of C_60_ Fullerene. Biotechnol. Appl. Biochem..

[46] Lazareva P.I., Stupin V.A., Lazarev K.A., Litvitskiy P.F., Manturova N.E., Silina E.V. (2025). Biodistribution and Toxicological Impact Assessment of Cerium Dioxide Nanoparticles in Murine Models: A Systematic Review of In Vivo and Ex Vivo Studies. Pharmaceutics.

[47] Su L.C., Xie Z., Zhang Y., Nguyen K.T., Yang J. (2014). Study on the antimicrobial properties of citrate-based biodegradable polymers. Front. Bioeng. Biotechnol..

[48] Azhogina T., Sazykina M., Konstantinova E., Khmelevtsova L., Minkina T., Antonenko E., Sushkova S., Khammami M., Mandzhieva S., Sazykin I. (2022). Bioaccessible PAH Influence on Distribution of Antibiotic Resistance Genes and Soil Toxicity of Different Types of Land Use. Environ. Sci. Pollut. Res..

[49] Prazdnova E.V., Chistyakov V.A., Churilov M.N., Mazanko M.S., Bren A.B., Volski A., Chikindas M.L. (2015). DNA-Protection and Antioxidant Properties of Fermentates from *Bacillus amyloliquefaciens* B-1895 and *Bacillus subtilis* KATMIRA1933. Lett. Appl. Microbiol..

[50] Milenković I., Radotić K., Despotović J., Lončarević B., Lješević M., Spasić S.Z., Nikolić A., Beškoski V.P. (2021). Toxicity Investigation of CeO_2_ Nanoparticles Coated with Glucose and Exopolysaccharides Levan and Pullulan on the Bacterium Vibrio Fischeri and Aquatic Organisms Daphnia Magna and Danio Rerio. Aquat. Toxicol..

[51] Cheng C., Cheng Y., Zhao S., Wang Q., Li S., Chen X., Yang X., Wei H. (2022). Multifunctional Nanozyme Hydrogel with Mucosal Healing Activity for Single-Dose Ulcerative Colitis Therapy. Bioconjug. Chem..

[52] Zhu W., Wang L., Li Q., Jiao L., Yu X., Gao X., Qiu H., Zhang Z., Bing W. (2021). Will the Bacteria Survive in the CeO_2_ Nanozyme-H_2_O_2_ System?. Molecules.

[53] Zamani K., Allah-Bakhshi N., Akhavan F., Yousefi M., Golmoradi R., Ramezani M., Bach H., Razavi S., Irajian G.-R., Gerami M. (2021). Antibacterial Effect of Cerium Oxide Nanoparticle against *Pseudomonas aeruginosa*. BMC Biotechnol..

[54] Surendra T.V., Roopan S.M. (2016). Photocatalytic and Antibacterial Properties of Phytosynthesized CeO_2_ NPs Using *Moringa oleifera* Peel Extract. J. Photochem. Photobiol. B Biol..

[55] Barker E., Shepherd J., Asencio I.O. (2022). The Use of Cerium Compounds as Antimicrobials for Biomedical Applications. Molecules.

[56] Dar M.A., Gul R., Alfadda A.A., Karim M.R., Kim D.W., Cheung C.L., Almajid A.A., Alharthi N.H., Pulakat L. (2017). Size-Dependent Effect of Nanoceria on Their Antibacterial Activity Towards *Escherichia coli*. Sci. Adv. Mater..

[57] Butt A., Ali J.S., Sajjad A., Naz S., Zia M. (2022). Biogenic Synthesis of Cerium Oxide Nanoparticles Using Petals of Cassia Glauca and Evaluation of Antimicrobial, Enzyme Inhibition, Antioxidant, and Nanozyme Activities. Biochem. Syst. Ecol..

[58] Bhargava N., Shanmugaiah V., Saxena M., Sharma M., Sethy N.K., Singh S.K., Balakrishnan K., Bhargava K., Das M. (2016). Nanocerium Oxide Increases the Survival of Adult Rod and Cone Photoreceptor in Culture by Abrogating Hydrogen Peroxide-Induced Oxidative Stress. Biointerphases.

[59] Del Turco S., Cappello V., Tapeinos C., Moscardini A., Sabatino L., Battaglini M., Melandro F., Torri F., Martinelli C., Babboni S. (2022). Cerium Oxide Nanoparticles Administration during Machine Perfusion of Discarded Human Livers: A Pilot Study. Liver Transpl..

[60] Elmorshdy S.E.E.M., Shaker G.A., Eldken Z.H., Salem M.A., Awadalla A., Shakour H.M.A., Sarhan M.E.E.H., Hussein A.M. (2023). Impact of Cerium Oxide Nanoparticles on Metabolic, Apoptotic, Autophagic and Antioxidant Changes in Doxorubicin-Induced Cardiomyopathy: Possible Underlying Mechanisms. Rep. Biochem. Mol. Biol..

[61] Dhall A., Self W. (2018). Cerium Oxide Nanoparticles: A Brief Review of Their Synthesis Methods and Biomedical Applications. Antioxidants.

[62] Singh S., Dosani T., Karakoti A.S., Kumar A., Seal S., Self W.T. (2011). A Phosphate-Dependent Shift in Redox State of Cerium Oxide Nanoparticles and Its Effects on Catalytic Properties. Biomaterials.

[63] Sassanfar M., Roberts J.W. (1990). Nature of the SOS-inducing signal in *Escherichia coli*: The involvement of DNA replication. J. Mol. Biol..

[64] Singh R., Wiseman B., Deemagarn T., Jha V., Switala J., Loewen P.C. (2008). Comparative study of catalase-peroxidases (KatGs). Arch. Biochem. Biophys..

[65] Amabile-Cuevas C.F., Demple B. (1991). Molecular characterization of the soxRS genes of *Escherichia coli*: Two genes control a superoxide stress regulon. Nucleic Acids Res..

[66] Gregor C., Gwosch K.C., Sahl S.J., Hell S.W. (2018). Strongly Enhanced Bacterial Bioluminescence with the *Ilux* Operon for Single-Cell Imaging. Proc. Natl. Acad. Sci. USA.

[67] Cormack B.P., Valdivia R.H., Falkow S. (1996). FACS-Optimized Mutants of the Green Fluorescent Protein (GFP). Gene.

[68] Shemiakina I.I., Ermakova G.V., Cranfill P.J., Baird M.A., Evans R.A., Souslova E.A., Staroverov D.B., Gorokhovatsky A.Y., Putintseva E.V., Gorodnicheva T.V. (2012). A Monomeric Red Fluorescent Protein with Low Cytotoxicity. Nat. Commun..

[69] Malke H. (1984). T. Maniatis, E. F. Fritsch and J. Sambrook, Molecular Cloning: A Laboratory Manual, X + 545 S., 61 Abb., 28 Tab. Cold Spring Harbor, N.Y. 1982. Cold Spring Harbor Laboratory. J. Basic Microbiol..

[70] Wiedemann B. (2006). Test results: Characterising the antimicrobial activity of daptomycin. Clin. Microbiol. Infect..

[71] Eltzov E., Ben-Yosef D.Z., Kushmaro A., Marks R. (2008). Detection of sub-inhibitory antibiotic concentrations via luminescent sensing bacteria and prediction of their mode of action. Sens. Actuators B Chem..

